# What does the development of medical tourism in Barbados hold for health equity? an exploratory qualitative case study

**DOI:** 10.1186/s41256-017-0025-z

**Published:** 2017-02-20

**Authors:** Ronald Labonté, Vivien Runnels, Valorie A. Crooks, Rory Johnston, Jeremy Snyder

**Affiliations:** 1 Globalization and Health Equity Research Unit, Faculty of Medicine, University of Ottawa, 850 Peter Morand Crescent, Ottawa, ON K1G 5Z3 Canada; 2 Department of Geography, Simon Fraser University, 8888 University Drive, Burnaby, BC V5A 1S6 Canada; 3 Faculty of Health Sciences, Simon Fraser University, 8888 University Drive, Burnaby, BC V5A 1S6 Canada

**Keywords:** Medical tourism, Health equity, Population health, Barbados, Access to health care

## Abstract

**Background:**

Although the global growth of privatized health care services in the form of medical tourism appears to generate economic benefits, there is debate about medical tourism’s impacts on health equity in countries that receive medical tourists. Studies of the processes of economic globalization in relation to social determinants of health suggest that medical tourism’s impacts on health equity can be both direct and indirect. Barbados, a small Caribbean nation which has universal public health care, private sector health care and a strong tourism industry, is interested in developing an enhanced medical tourism sector. In order to appreciate Barbadians’ understanding of how a medical tourism industry might impact health equity.

**Methods:**

We conducted 50 individual and small-group interviews in Barbados with stakeholders including government officials, business and health professionals. The interviews were coded and analyzed deductively using the schedule’s questions, and inductively for novel findings, and discussed by the authors.

**Results:**

The findings suggest that in spite of Barbados’ universal health care and strong population health indicators, there is expressed concern for medical tourism’s impact on health equity. Informants pointed to the direct ways in which the domestic population might access more health care through medical tourism and how privately-provided medical tourism in Barbados could provide health benefits indirectly to the Barbadian populations. At the same time, they cautioned that these benefits may not materialize. For example, the transfer of public resources — health workers, money, infrastructure and equipment — to the private sector to support medical tourism with little to no return to government revenues could result in health inequity through reductions in access to and availability of health care for residents.

**Conclusions:**

In clarifying the direct and indirect pathways by which medical tourism can impact health equity, these findings have implications for health system stakeholders and decision-makers in Barbados and other countries attempting both to build a medical tourism industry and to protect health equity.

## Background

Medical tourism describes the pursuit of non-emergent or elective medical care outside of one’s own country for which an individual pays privately. There are many reasons why individuals choose to pursue such care abroad, which have been well-described elsewhere [1–3]. Our focus in this article is on how different stakeholders in Barbados, a country with a well-developed tourism industry and evidence of sustained efforts to develop health service exports, perceive the anticipated health equity impacts of an enhanced medical tourism sector [4–7]. Although medical tourism exists in many countries, evidence of where it impacts health systems and with what health equity effects is largely lacking [8–10].

A small country with a 2015 population of 290,604 [11], Barbados is considered a high income country although its gross national income (GNI) per capita in 2014 was at the lowest end of the ‘high income country’ designation [12]. Its income Gini coefficient, an expression of income inequality,^1^ is also high at 0.47 [13], with several United Nations agencies considering a coefficient above 0.4 to be extreme, with the risk of creating social instability [14]. Barbados spends more on health care that any other country in the Caribbean and Central American region [15] in a mixed public/private system. Approximately 65% of health care is public and 35% private, with up to 80% of private health care paid for out-of-pocket [16]. The public health system is universally accessible, free at the point of delivery, and comprehensive including access to drugs through the Barbados Drug Service. Access to the public health system is limited by relatively longer waiting times than in the private clinics [17]. Access to privatized health services by the poor is limited because of cost.

Barbados is one of many countries in the Latin American/Caribbean (LAC) region attempting to develop an expanded medical tourism sector to generate foreign currency earnings, create new employment opportunities and improve the range and quality of health care for local residents. Its current medical tourism industry is described as “modest in size and significance relative to those that exist in other Caribbean nations” [5]. Barbados is also one of three countries in the LAC region involved in our comparative research on medical tourism, the other two being Guatemala and Mexico. While Mexico has an already well-established medical tourism industry, both Guatemala and Barbados are in the early stages of developing this sector, providing an opportunity to explore *ex ante* and in depth how different stakeholder groups perceive its health equity implications.

Our understanding of health equity is based on Braveman and Gruskin’s definition as “the absence of systematic disparities in health (or in the major social determinants of health) between social groups who have different levels of underlying social advantage/disadvantage and in its key social determinants) that are systematically associated with social advantage/disadvantage” [18] p. 254. In brief, health equity incorporates notions of social justice and fairness and is determined by who gets what, when and how in health care [19, 20].

How health equity can be understood in relation to medical tourism is associated closely with how a fair distribution of health and access to health care in a population is achieved, and how medical tourism might affect this. Medical tourism, in theory, can directly or indirectly improve the health of the local (domestic) population [21]. Directly, any increases in infrastructure, facilities and health human resources associated with medical tourism could be made accessible to some or all local citizens, or help to relieve access pressures on public health systems. Indirect health improvement can take place through the provision of jobs in medical tourism for otherwise unemployed health and ancillary workers, employment being an important determinant of health. Population health may also benefit indirectly from business and income tax revenues from medical tourism that governments use to fund health care and other social determinants of health [22, 23]. Some scholars and policy experts have raised concerns with the distribution of these theorized benefits, noting that medical tourism is unlikely to provide direct or demonstrable indirect benefits to domestic populations and is more likely to threaten health equity in terms of access to health care (whether publicly or privately provided) [22, 24].

Existing medical tourism literature captures some of the pathways by which medical tourism might impact health equity [5, 25–28]. In this paper we use Barbados as a single case country to identify and articulate these pathways in greater detail. The resulting analysis provides a set of useful and transferable decision-making points governments should consider to mitigate any potential negative health equity impacts arising from development or growth of medical tourism.

## Methods

We conducted and analyzed a series of individual and small-group interviews^2^ in Barbados (*N* = 50) to identify if and how medical tourism might affect health equity in Barbados. The interview schedules made use of a background literature review that was developed to characterize the health care and medical tourism environment in Barbados and identify issues to be probed in the interviews (4). The recruitment strategy was designed to capture a range of views on medical tourism through a variety of engaged stakeholders (15 each from public health/tourism sector, private health/tourism sector and health human resources sector, and 5 from civil society). Participants from the public sector were those in policy development or analyses related to medical tourism or trade and health; from the private/tourism sector private facility administrators, tourism operators and medical tourism consultants; from health human resources those involved in training, certification and regulation as well as front-line service delivery; and from civil society those active in non-governmental organizations and health journalists/academics. Participants were identified through secondary data collected on medical tourism, by roles within public and private sector organizations, and through a rolling (‘snowball’) recruitment from interviewees. Interviews were subject to the informed consent of the participants and received approval from the ethics boards at Simon Fraser University (certificate number 2012 s0148), the University of Ottawa (certificate number 1104-12-13), and the University of the West Indies, Cave Hill. Interviews took place over a period of 6 weeks in the summer of 2013 and 2 weeks in February 2014. Interviews typically lasted between 45 and 90 min, and were semi-structured to allow both the interviewer and participant to pursue clarification and certain lines of discussion. Interviews were entered into NVivo, a qualitative data analysis software,^3^ and coded deductively using the schedule’s questions, and inductively for novel findings. The coding and analysis were discussed by the group of authors. Because Barbados is such a small island, we have not used identifiers with interview quotes in reporting the results with the exception of a letter and number associated with the informant, in order to protect their identities.

We established qualitative rigour in this study in multiple ways: we employed investigator triangulation by seeking confirmation of interpretation of the findings between multiple researchers; we established an audit trail by keeping detailed records that document not only the process of data collection but also key decisions made throughout the study; we provided contextual information about the study site and the participants making it possible for others to assess the potential for transferability of the findings to other contexts; and we led dissemination events within the country that offered study participants (and others) to pose questions, raise concerns or offer elaborations upon our own interpretations of the data. These efforts increase the trustworthiness of our results.

For this article, we also collected recent Barbadian social and economic indicators suggestive of health equity/inequity, including measures indicative of access to care, such as primary health care coverage across the population.

## Results

As for many countries, medical tourism in Barbados is seen as one opportunity for economic development and growth. Positive factors for its development include low corruption, high literacy, and high value-added products [15, 29] and that Barbados is proximate to the US, less than 4 h from Miami, Florida, by plane. The US is considered to be a large potential market for ‘medical tourists,’ making its capture by countries competing for such patients highly desirable [26, 27]. Barbados is also considered a mature tourist destination accessible to Canada, the United Kingdom and the European region. Along with the US, these countries already provide large numbers of holiday visitors to the Caribbean, further suggesting substantial potential markets for medical tourism [30]. There is also significant arranged cross-border care in this area of the Caribbean [31].

Although Barbados appears to be well-positioned to participate in medical tourism, there is presently only limited evidence of medical tourism in Barbados, consisting primarily of a well-known fertility clinic that, as one respondent reported, is situated “right by the beach… (where) you can hear the waves and you come for your treatment but then you can actually also just stay at the hotel down the road which is right on the beach and it’s so relaxing” (Participant G9).^4^ An expansion of treatments currently available in health and wellness spas (to include surgical “nips and tucks,” “medical procedures done at the high end spas,” and an interest in “broaden(ing) their product range by involving medical doctors at a deeper degree” (Participant G9)) is seen as one route towards building medical tourism in Barbados. In addition to a model of Barbados-based and regulated health care facilities (such as the existing fertility clinic), there was frequent reference by respondents to the planned development of stand-alone foreign-owned and operated health facilities. Respondents reported ongoing discussion about the development of the former St. Joseph public hospital site by the US-led Traverse Global Healthcare group [6]. This facility would mostly target medical tourists from the US and be staffed largely by part-time US-based physicians, suggesting a stand-alone model of medical tourism that would not rely on the local population.

### Characterization of the Barbadian health care system and health equity

Respondents’ characterization of the Barbadian health care system is an important basis for interpreting local understanding of the health equity impacts of medical tourism. According to the respondents, the Barbadian health care system is built upon a principle of fairness for citizens. One respondent comprehensively described this perspective as “our whole thrust of equity and universal access and affordability and sustainability” (Participant H11). The government goal of improving health care in Barbados was described as “we have tried to develop health in Barbados to be better….to try to achieve some of the things that more developed countries have had” (Participant H11). In this context, ensuring that non-Barbadian medical tourists do not take advantage of the public system to the detriment of health equity was important to respondents. To that end, Barbadian health system stakeholders offered cautious support for medical tourism conditional on its demonstrated benefits to the country’s economy and health care system, while also ensuring high standards and quality of care, an area that appeared to require attention: “one of our biggest roadblocks right now, [is] development of standards… if that’s not done we really can’t move forward” (Participant H8).

### Potential models of medical tourism

Most respondents presumed that the country’s eventual medical tourism industry would be strictly stand-alone private health care (i.e. not partnered with the public sector) with no negative impact on public health care, “I don’t think there was ever a concern that …medical tourism would impact and deplete any of the local equipment or peripheral needs, but that it should bring stuff to the island” (Participant P9). All facilities, staff and equipment would be provided by the medical tourism clinic: “We would presume that (…) where there are patients coming for orthopaedic surgery that all the increased equipment needs would be brought in by that company” (Participant P9). This approach was extended to the ‘fly-in’ approach to medical tourism, in which the existing private sector in Barbados would not be implicated in any way with an offshore-type model where “models [being considered] would bring in their own physicians” (Participant P9).

If well regulated, privately provided medical tourism, as one respondent expressed, “could be beneficial to the public side” but only “as long as it is not affecting … my opportunity to seek my own health care in the country, and I am assured that what is being done is bringing revenue in which will perhaps improve my own health care eventually” (Participant H4). Relatedly:

… if people didn’t see [medical tourism] as a drain on the public health resources. If they saw it as something which was…adding revenue, adding income, then it wouldn’t be a problem. Because tourism is our main industry and…if we haven’t got tourism, we haven’t got a lot (Participant H1).

In other words, medical tourism was conceived of as a private sector activity with no actual involvement of public sector health care, but which could provide indirect benefit to government revenue and eventually improve population health equity. However, this perspective did not mean that public government was excluded from medical tourism; rather, its role was seen as enabling of, and critical to, the development of medical tourism.

### Government, health equity and medical tourism

Private sector enterprise respondents in particular commented on the potential benefits of using revenues from medical tourism to support the public health care system, thereby benefitting health equity indirectly via improved public health care for the population as a whole. However, respondents also reported concessions and ‘special’ incentive packages for foreign investments in the hotel industry, manufacturing and offshore business services; reduced corporate tax rates on income, gains and profits; and a Market Development Allowance to the tourism sector. All of these instruments would reduce the size of any government revenue from medical tourism [29].

Respondents pointed to a recent amendment of the Tourism Development Act which, as well as addressing income tax concessions and investment tax credits, deals with custom duty waivers on medical imports consumed by or provided for tourists [32]. The amendment was a contentious point among research participants as physicians not interested in treating foreigners thought themselves to be at a competitive disadvantage to those that did and who would be able to obtain duty waivers through this Act on medical equipment. Although this legislation is limited to providing concessions to the aforementioned fertility clinic, other respondents reported that concessions for medical imports were being permitted elsewhere.

Medical tourism was also thought to bring increased opportunities for health education and training. New medical tourism facilities could offer medical residents improved educational and training opportunities through exposure to different technologies and procedures than those presently offered in the public system (Participant P3). Training opportunities for health workers and hospital administrators could also be expanded (Participant P3), especially nurse training. Given the mobility and emigration of nurses from the Caribbean region, several respondents noted that improved nurse training and employment opportunities would lead to “people [nurses] staying on the island” and “that would be a bonus” (Participant H1). The existence of improved education and training would contribute to health equity by assisting with staff retention and the maintenance of population access to health care in the public sector.

### Health equity risks

That medical tourism is private health care *per se* (a dominant concern in much of the writing on health equity issues in the medical tourism literature) did not seem to be an issue for our study respondents [33]. Barbadians are covered by both a universal public health system as well as optional private health care for those that can afford it. Currently, the private/public mix is more heavily skewed towards maintaining public health care. Many doctors who work within both systems are required to work a certain number of hours in the public system (Participant H12) [34]. Some physicians work solely in the private sector, while others work on occasion on public cases in public sector facilities (Participant H12). Private patients still benefit from the public system, with informants providing evidence of transfers of private patients to the public care system at certain points in their care trajectories. Rather, the equity concern for our respondents was two-fold and revolved around the intersections and flow between the public and private systems.

The boundary between private medical tourism and public health care in Barbados is fuzzy. For example, as some respondents noted, there are still questions regarding use of the public blood supply in Barbados when medical tourists need transfusions or if public emergency care is needed, and/or how the provision of follow-up care is provided once medical tourists have left the private system yet remain in the country. These examples represent points at which public funds may be required to subsidize foreign patients. But this boundary is permeable in the other direction as well: public health patients can be transferred to the private sector, although in the case of medical tourism this is yet to be demonstrated. Further to this, there was worry that the private health care available only for foreign patients may differ in quality from that offered in the public and private sectors serving the local Barbadian population, with the implication that it would create “one set of [better] health care for the medical tourists, another set for the local population” (Participant H11).

There was some suggestion in the interviews that the local population would actually be unhappy with a wholly separate or stand-alone medical tourism model, such as the ‘fly-in’ model noted above. The public system, it was noted, has waiting lists for and delays in providing some surgeries, such as for total hip replacements which would likely be one of the services offered by medical tourism facilities. A wholly stand-alone model, apart from any tax revenues it generated, would not benefit the local population in that regard. Some interviewees thought that, should a private medical tourism facility benefit in any way from the public sector, it should also offer facilities and opportunities to relieve pressures on the public sector: “It’s the small private sector that is benefiting [from medical tourism], but still if it’s totally nothing … [that] filters down to the guy on the street, that would be a concern” (Participant P9). 5The same respondent went on to argue that “If local physicians had access to use that private facility for their own patients then that would be something that I think that local physicians would accept.” A government respondent echoed the same sentiment: “Should locals be interested [in medical tourism services] they should also have access…and [if] they can afford the services, they should not be excluded” (Participant G6). Regardless, the focus of the private/public sector interaction was often brought back to concern for the local population:

Private citizens recognize that medical tourism will be good for Barbados…it’s a good fit for the country … it will be good for the economy and we recognize there’s a need to diversify, and we’re always looking for new products and services that could help us to earn foreign exchange and to create employment for locals…but we also have to balance the need of the population as a whole (Participant G6).

Finally, and consistent with cautions echoed in the broader medical tourism literature, several respondents were troubled by the effect its development in Barbados might have in attracting away from the public sector the already limited supply of health workers in the country. Barbadian nurses in particular were seen as potentially mobile workers, dependent on the availability and quality of employment, and interested in better salaries and working conditions regardless of whether this would be in the private or public sectors. “If you get investors come in and they set up a new facility and they’re going to pay nurses more than the current, than anybody else on the island is paying, the nurses are going to go to that new facility” (Participant P9). Such a medical tourism facility could also attract nursing staff from outside Barbados, “If the facility is having an employment package that’s attractive, it’s not only attractive to Barbadians, but Jamaicans, any nurses in the Caribbean would travel to work…within the Caribbean there’s enough nurses moving around and nurses move to where they can get good pay and that’s just the way it is” (Participant P9).

### Government opportunity costs

Despite some suggestion that Barbadians are somewhat averse to “foreign money” which is “foreign administered” (Participant P3), due at least in part to the dominance of foreign investment and administration in the country’s tourism sector, the development of medical tourism in Barbados will almost certainly be financed externally (Participant H8). Foreign investment necessarily requires government involvement and approvals. Government, according to some respondents, has been “…a bit slow in developing new legislation [regarding medical tourism], and even when we start the process…it takes a little while for it to be enacted” (Participant G6). There were other complaints too about the slow pace of government decision-making with respect to opening a private medical tourism hospital (Participant H12), while others noted how changes in health ministry leadership have been “one of the hindrances to the thing [sector development] moving forward” (Participant P9).

This slow pace may be due in part to a lack of enthusiasm for innovation in the health sector, “the government does not really encourage you to go ahead and do this, we have to push it” (Participant H12). Nonetheless, and consistent with findings from other studies, the Barbados government is anticipating a significant inflow of foreign exchange primarily from foreign patients coming to Barbados [35]. This expectation had some respondents noting the concessions to tourism the government already makes, calling on the government to be “a bit more flexible and introduce some other concessions to help the [medical tourism] sector to grow” (Participant G6). A regional backdrop to the lack of momentum could well be the extent of regional competition for medical tourism, the country’s perceived lack of cost advantage (Participant H12), and the poor profitability of its own private care facilities (Participant P3).

Other Barbadian government cost concerns noted by respondents included indirect costs associated with a need to increase capacity for physician registration, regulation to manage increased numbers of health workers and accreditation of facilities (Participant P9). Issues regarding regulation of malpractice insurance of health workers providing medical tourists with care were also raised (Participant P9).

Medical tourism agreements and partnerships between the government and private medical tourism developers surface possible conflicts of interest as governmental alignment with the private sector has potential to conflict with the government’s roles in serving the welfare of the citizens of Barbados. For example, Invest Barbados’ request for proposals for the development of St. Joseph Hospital resulted in the hospital site’s acquisition by American World Clinics (now Traverse Global Healthcare), a private company. One respondent stated, “… (government) are just interested in providing the facility and the land as a landlord [which] is to be leased to American World Clinics, but they’re not going to be actively involved in running the business or anything” (Participant G9), suggesting a wholesale transfer of the facility to the private sector and a loss of role for the government in directing a portion of health care for the public benefit in Barbados.

## Discussion

At the beginning of this paper, we proposed that medical tourism can influence health equity in two main ways — directly through health care systems and improved (or worsened) access to health care for provider countries’ populations, and indirectly through, for example, employment opportunities and/or government revenues invested in both public health care and social determinants of health. Our specific focus and findings in this paper on Barbados suggest that both types of pathways are operational.

### Direct and indirect pathways to health equity

If medical tourism is to bring improvement to health equity in Barbados, it must be able to offer direct services or opportunities that will increase health care access to domestic populations or alternatively (and indirectly) increase access to social determinants of health that results in improvement to population health status. Respondents first recognized medical tourism as private sector health care with potential negative impacts on provision of care in the public health sector, particularly through the movement of public sector employees to the private sector, depleting human resources in the public sector [5]. Attracting workers to Barbados’ medical tourism sector from other parts of the Caribbean where shortages exist also raises the issue of potentially increasing health inequity not only within but also between Caribbean countries, and opportunity costs incurred to train and replace missing staff.

The Barbadian government’s roles in developing medical tourism are not only key but wholly linked to the protection, maintenance and/or generation of health equity. There was indication (and implied critique) of the government’s slow response to the development of legislation supporting medical tourism. There was suggestion, although less direct, of government’s concern with costs implicated with medical tourism. Any medical tourism development in Barbados would require government investment (e.g., with regard to regulation and system support) thereby incurring some level of public costs that, at least in the short term, could represent foregone public investments in domestic public health. Government investment/public costs could however be insubstantial and could be offset by policies ensuring a reasonable public revenue capture of medical tourism’s profits.

Respondents highlighted the government’s multiple roles in medical tourism, as health regulator, promoter and tax collector, as well as functioning as public health care provider. This multiplicity also suggests that it will be a challenge to avoid conflicts of interest given that the government will be simultaneously in a position of incentivizing as well as regulating the medical tourism industry in Barbados.

These views about compromised health access and health equity were partly dependent on the type of medical tourism model being pursued, whether using Barbados’ public or private health care facilities or stand-alone models. Some respondents favoured a separate or stand-alone health care system, isolated from existing public and private health care facilities and insulated from any potential negative impacts on the public health care system. Others questioned whether this was possible or even desirable, especially given the country’s already mixed public/private system, and the potential of public care to piggy-back on the private sector. Local physician support for medical tourism may be dependent on access to new facilities for their own private patients, although this could exacerbate access inequalities between those with the ability to pay and those reliant on the public system. Increasing direct access to health care for poor or non-insured populations, while on paper appearing to generate benefits in health equity for patients served, was barely considered as a health equity benefit of private sector medical tourism in Barbados.

Some countries (e.g., India and Mexico) have attempted to mitigate inequity somewhat by requiring a percentage of services and bed occupancies in private hospitals (whether or not they are medical tourism-only facilities) to local non-paying indigent patients in return for land or tax concessions, although these policies have had generally poor results in serving the public due to non-compliance by private actors and poor government enforcement [36, 37]. However, a recent study of medical tourism in Thailand estimated that the knock-on benefits of the non-medical ‘tourism’ expenditures of patients and families were substantial [38]. If these non-medical profits were similarly taxed for public health care re-investment, there is little reason to preclude the possibility of medical tourism revenues in Barbados indirectly generating a net health equity gain. Much will depend on the regulatory approach taken by the Barbadian government, as countries such as India and Colombia promote their medical tourism facilities, in part, through large-scale tax concessions with foregone public revenue implications [39–42].

Benefits to health equity of stand-alone models of medical tourism were harder to demonstrate from the study participants’ perspective. In the case of concessions to medical tourism operators, respondents indicated that amendments to the Tourism Development Act were broadly interpretable, suggesting that potential revenue streams would be diminished with some (albeit likely small) losses in potential revenue for public health or other public investments in social determinants of health. Tourism activities related to medical tourism is a further source of health equity benefits, to the extent that revenues are equitably distributed and taxes on tourism earnings are invested in public health or improvements in social determinants of health. Training opportunities potentially afforded by the (presumed) new medical technologies that medical tourism facilities would bring to Barbados were also seen as health equity positives. Concerns over impacts — specifically depletion — on health human resources appeared the most cogent in terms of health equity risks to the public health care system, less so for physicians (assuming that the ‘fly-in’ models seen with Traverse Global Healthcare predominate) but more so for nurses, which also has regional health equity implications.^6^ At present, these are only hypothetical concerns but they are supported by findings of ‘internal brain drain’ from public to private facilities in other studies [43].

In summary, medical tourism in Barbados could offer a number of positive benefits for health equity, including through: improved opportunities for medical and nursing education having indirect impact on raising the quality of public health care; revenue to governments (taxation can be used directly on increasing access to care and improving population health); and the possibility of public sector patient transfers to private care where public care is insufficient thereby increasing access to health care. But there are health equity risks, too, associated with how the profit-focused goals of private industry might undermine those of equity-based public health care.

Drawing from the insights of Barbadian stakeholders contemplating development of medical tourism within their country, the following key points for a health-equitable approach emerge:

- Public revenues (from providing medical care to foreign patients or from associated non-medical expenditures in the country) should exceed substantively any public costs (promotion, regulation, concessions, training) associated with developing the industry.
- Publicly stated promises, potentially even legislative commitments, to using new public revenues to strengthen the local public health system should precede any final decisions on granting permissions to open a medical tourism facility.
- Policies should be in place to allow local access to services in such a facility on either a privately paid basis or through a percentage allocation of public health care patients.
- If access to such facilities is on a privately paid basis, policies and public expenditures should ensure that the overall proportion of health care resources available to both private and public patients is roughly equal and needs-based.
- Regulations governing employment conditions should ensure an equitable distribution of health human resources (based on the population size and need) across public and private health care sectors.
- Routine economic and health system assessments should be undertaken to ensure that medical tourism (with a full accounting of its direct and indirect benefits and costs) is creating a net benefit equitably distributed across the local population.

## Conclusions

Although data remain imprecise, there is a general consensus in the literature that medical tourism is likely to continue to grow. Many countries across the development scale (from low— to middle— and high-income) are also likely to continue seeking foreign currency earnings by providing services to international fee-paying patients. The implicit health equity risk is the extent to which such private-sector led growth crowds out access for local populations, especially poorer sectors of those populations. The existing health care system,and its regulatory and financing structures, are important enablers or constraints on medical tourism’s ability to improve health equity.

The concluding points from our Barbados case study are likely transferable across many countries contemplating development or growth of a medical tourism sector, some irrespective of existing health system organization since they are premised on movement towards a more health equitable outcome. Undertaking an assessment along such lines prior to medical tourism development or scale-up, and incorporating within it qualitative inquiry such as that undertaken in the Barbados study, could contribute to creating a regulatory and policy environment that maximizes population health equity gains. Doing so would also be consistent with the government commitments to the 2015 Sustainable Development Goals, notably the target to achieve Universal Health Coverage: “…that all people can use the promotive, preventive, curative, rehabilitative and palliative health services they need, of sufficient quality to be effective, while also ensuring that the use of these services does not expose the user to financial hardship” [44].

## Abbreviations

GNI: Gross national income
HDI: Human development index
HIV/AIDS: Human immunodeficiency virus/acquired immune deficiency syndrome
PPP: Purchasing power parity
US: United States
USD: American dollars ($)

## Footnotes

1 A measure of the deviation of the distribution of income among individuals or households within a country from a perfectly equal distribution. A value of 0 represents absolute equality, a value of 100 absolute inequality. Source: http://hdr.undp.org/en/content/income-gini-coefficient Accessed September 12 2016.

2 Small group interviews were conducted only when requested by participants, and only with people with an established history of working together.

3 NVivo qualitative data analysis Software; QSR International Pty Ltd. Version 10, 2012.

4 There is also a cardiac facility located in Bridgetown that treats international patients experiencing cardiac emergencies, and a dialysis clinic that treats vacationing tourists. Neither of these constitute a medical tourism facility in the strict sense of international patients specifically traveling to them for care. There was also an earlier effort (between 2004 and 2010) to create a new private hospital primarily for domestic patients, with management by the Indian-based Apollo Hospital chain. Unlike the Barbadian hospital developers, the Apollo chain wanted the facility to target principally foreign American medical tourists. The disagreement led to abandonment of the initiative.

5 We note that regardless of what model of medical tourism Barbados eventually pursues, the country’s sole (public) tertiary hospital would inevitably be a default provider for any emergencies or limitations experienced by the private medical tourism system, assuming some proportion of the treatment costs.

6 In footnote 2 we referenced some limited development of a proposal between 2004 and 2010 to create a hospital in Barbados in conjunction with the Indian-based Apollo group. This was a privately-led initiative and the planning did not get far enough along for us to be able to comment on proposed staffing models. As such, it is not possible to connect this proposal to a discussion of the implications for a fly-in model of staffing/care provision.
